# Multisite biologic tissue SARS-CoV-2 PCR testing in kidney transplantation from a COVID-positive donor

**DOI:** 10.1093/jscr/rjac314

**Published:** 2022-07-05

**Authors:** Jared R Zhang, Michael Kueht, A Scott Lea, Heather L Stevenson, Joseph Gosnell, Ping Ren, Marisa C Nielsen, Aaron Miller, Muhammad Mujtaba, Jeffrey Fair

**Affiliations:** UTMB, Department of Surgery, Division of Transplant Surgery, University Boulevard, Galveston, Texas, USA; UTMB, Department of Surgery, Division of Transplant Surgery, University Boulevard, Galveston, Texas, USA; UTMB, Department of Medicine, Division of Infectious Diseases, University Boulevard, Galveston, Texas, USA; UTMB, Department of Pathology, University Boulevard, Galveston, Texas, USA; UTMB, Department of Pathology, University Boulevard, Galveston, Texas, USA; UTMB, Department of Pathology, University Boulevard, Galveston, Texas, USA; UTMB, Department of Pathology, University Boulevard, Galveston, Texas, USA; UTMB, Department of Pediatrics, Vaccinology, University Boulevard, Galveston, Texas, USA; UTMB, Department of Medicine, Division of Transplant Nephrology, University Boulevard, Galveston, Texas, USA; UTMB, Department of Surgery, Division of Transplant Surgery, University Boulevard, Galveston, Texas, USA

## Abstract

With a high community transmission rate, SARS-CoV-2 has profoundly exacerbated the shortage of organs. Although the risk of donor-recipient transmission of SARS-CoV-2 is anecdotally low, an organ-specific infection analysis of procured organs from SARS-CoV-2 positive donors has yet to be established. Using a combination of clinically available and research-only polymerase chain reaction methods, organ preservation fluid and renal parenchymal tissues were tested for SARS-CoV-2 from the kidney of a SARS-CoV-2-positive donor prior to transplantation. The recipient has remained SARS-CoV-2 negative and clinically well, with excellent graft function 120 days post-transplantation.

## INTRODUCTION

Due to the ubiquitous prevalence and myriad of clinical presentations of COVID-19, multiple guideline-issuing societies have proposed approaches to SARS-CoV-2-positive organ donors [1, 2]. As transplant recipients are inherently immunosuppressed, higher risks of COVID-19 infection coupled with higher morbidity and mortality risks have been identified [3]. Therefore, heightened concerns exist over donor–recipient transmission of SARS-CoV-2.

Currently, there is no consensus regarding the evaluation of organs procured from positive donors. Although multiple case reports have shown acceptable clinical outcomes in recipients of non-lung transplantations from SARS-CoV-2-positive donors, testing modalities remain markedly heterogeneous and occasionally lacking [4]. To expand the pool of eligible organ donors, it is imperative to identify through coherent testing strategies, the clinical scenarios associated with low disease transmission risk.

We describe the testing strategies used for successful kidney transplantation from a SARS-CoV-2-positive deceased donor. Besides donor nasopharyngeal (NP) swabs and lower respiratory tract samples, organ preservation fluid and renal tissue from the allograft were tested.

## CASE REPORT

The donor was an ABO blood type B teenage male who succumbed to head trauma. Twenty-three days previous to presentation, the patient tested positive for SARS-CoV-2 via an NP swab (testing specifics unavailable). No respiratory complaints were reported by the patient. Cross-sectional chest imaging revealed air bronchograms and patchy ground glass opacities in the left lower lobe and right upper, middle and lower lobes. The etiologies of infection and inflammation were differential. Oxygenation was moderately impaired (PaO_2_:FiO_2_ = 290).

On day 3 post-admission, donor bronchoalveolar lavage (BAL) specimens tested negative for SARS-CoV-2 (Panther Fusion® System SARS-CoV-2 Assay) (Table 1) [5]. However, on the same day, an NP swab tested positive (Alinity m SARS-CoV-2 Assay, Abbott) [6].

**Table 1:** SARS-CoV-2 testing methods, mechanism, targets and results of donor

**DONOR**	**9/17/21**	**9/19/21**		**9/20/21**		**9/25/21**	
**Specimen type**	NP swab	NP swab	BAL	NP swab	BAL	Kidney perfusion fluid	Kidney parenchyma
Test method	*	Abbott Alinity m Assay	Panther Fusion	Abbott Alinity m Assay	Panther Fusion	Cepheid Xpert Xpress	Novaplex SARS-CoV-2 Variants I, II, IV Assays
Test mechanism	PCR	RT-PCR	RT-PCR	RT-PCR	RT-PCR	RT-PCR	RT-PCR
Target	*	RdRp; N-genes	ORF1ab	RdRp; N-genes	ORF1ab	N2; E	RdRp; HV69/70 del, W152C, K417N, K417T, L452R, E484K, N501Y, P681R
Result	Negative	**Positive**	Negative	**Positive**	Negative	Negative	Negative

^*^Outside hospital-developed COVID PCR – no details available.

**Abbreviation**: NP swab: nasopharyngeal swab (upper respiratory tract); BAL: bronchoalveolar lavage (lower respiratory tract); RT-PCR: reverse transcription polymerase chain reaction; RdRp: RNA-dependent RNA Polymerase; HRP: horse radish peroxidase; IgG: immunoglobulin type G; ORF1ab: open reading frame 1 ab.

Organ procurement followed the standard protocol including rapid cooling, total body exsanguination and transfusion with four liters of cold Belzer UW solution (Bridge to Life Ltd., Northbrook, IL, USA). After procurement, the kidney allograft was stored in Custodiol HTK solution (Essential Pharmaceuticals, LLC, Durham, NC, USA) and transported using hypothermic static storage at ~2°C [7, 8]. The total cold ischemic time was 14 h, 22 min.

Prior to implantation, the preservation fluid tested negative for SARS-CoV-2. Briefly, a 10-cc aliquot of preservation fluid was tested, which the kidney had spent the previous 14 h in. This was analyzed using the Xpert® Xpress SARS-CoV-2 assay (Cepheid GeneXpert® Infinity System, Cepheid; RT-PCR) [9].

A 20-gauge parenchyma tissue biopsy sample (~50 mg) was obtained from the upper pole of the kidney allograft and immediately sent to the laboratory. RNA was extracted from the tissue using the PureLink™ FFPE Total RNA Isolation Kit (Invitrogen, Carlsbad, CA, USA). Extracted RNA samples (5 μl) were used to perform Novaplex SARS-CoV-2 Variants I, II and IV Assays (Seegene Technologies, Walnut Creek, CA, USA) to detect and confirm the SARS-CoV-2 variant, assessing for the alpha, beta, delta and omicron variants [10]. No SARS-CoV-2 was detected.

Five days following the donor’s last COVID test, transplantation proceeded uneventfully into a 34-year-old female experiencing end-stage renal disease secondary to systemic lupus erythematosus. This patient was an ABO blood type, had no history of COVID-19 infection and had received a second dose of an anti-SARS-CoV-2 vaccination (BNT162b2, Pfizer) four months previous. The recipients serum tested positive for anti-SARS-CoV-2 IgG (VITROS Immunodiagnostic Products Anti-SARS-CoV-2 test) (Table 2) [11].

**Table 2:** SARS-CoV-2 testing methods, mechanism, targets and results of recipient

**Recipient**	**9/25/21** ^**^			**9/29/21**
**Specimen type**	NP swab	NP swab	Serum	NP swab
Test method	Abbott Rapid ID NOW	Cepheid Xpert Xpress	VITROS Anti-SARS-CoV-2 Serology	Abbott Rapid ID NOW
Test mechanism	Isothermal nucleic acid amplification	RT-PCR	Qualitative chemiluminescent (HRP ‘sandwich’) immunoassay	Isothermal nucleic acid amplification
Target	Section of RdRp	N2; E	Anti-spike protein S1.10 IgG	Section of RdRp
Result	Negative	Negative	**Positive**	Negative

^**^Date of transplant

**Abbreviation**: NP swab: nasopharyngeal swab (upper respiratory tract); BAL: bronchoalveolar Lavage (lower respiratory tract); RT-PCR: reverse transcription polymerase chain reaction; RdRp: RNA-dependent RNA polymerase; HRP: horse radish peroxidase; IgG: immunoglobulin type G.

On the day of transplantation, an NP sample from the recipient tested negative for COVID using isothermal amplification (Abbott ID NOW, Abbott; target: RdRp) and reverse transcription polymerase chain reaction (RT-PCR) assays (Cepheid Xpert Xpress SARS-CoV-2 NAAT assay) [9, 12, 13].

An NP recipient swab tested negative 4 days postoperatively (ID NOW). No symptoms consistent with those of COVID-19 were observed. Normal allograft function was immediate, and serum creatinine levels decreased appropriately. Immunosuppression was induced using thymoglobulin and a high-dose of solumedrol and maintained with a triple-drug regimen (mycophenolic acid, prednisone and tacrolimus). At 120 days post-transplantation, the recipient was clinically well with excellent graft function (serum creatinine: 0.78 mg/dL).

## DISCUSSION

The pandemic has had a profound impact on millions of people globally, causing the number of transplantable solid organs to plummet dramatically [14]. Considering the high community transmission rates, it is imperative to develop detailed selection criteria for organ procurement from SARS-CoV-2-positive donors to increase the availability of transplantable organs. Recently, a summary from the Ad Hoc Disease Transmission Advisory Committee of Organ Procurement and Transplantation Network stated that donors with resolved COVID-19 are unlikely to transmit infection to non-lung recipients; however, due to the limited sample size of published studies, the exact transmissibility of SARS-CoV-2 from donors with active infection to non-lung recipients remains unknown [2].

In this report, we present a successful 120-day outcome of a kidney transplant from a SARS-CoV-2-positive deceased donor, in which NP swab samples, perfusion fluid and donor organ tissue biopsies were prospectively tested for SARS-CoV-2 (Table 1, Table 2).

Due to the limited number of reported cases, the prognosis of transplantation from SARS-CoV-2-positive donors remains poorly defined. Of the 13 reported clinical cases of successful transplantation from SARS-CoV-2-positive donors, specimens from the upper and lower respiratory tracts (NP and BAL) were commonly tested; however, data regarding organ-specific infection status were rarely reported and tended to be retrospective.

Perfusion fluid plays a vital role in organ preservation, spending long durations in contact with allografts to ensure metabolite clearance [15]. Therefore, perfusion fluid analysis could provide key information for understanding the infection status of donor organs and function as a potential marker for SARS-CoV-2 transmission risk.

In this case, we monitored the virological status of SARS-CoV-2 in the preservation fluid and parenchyma tissue of the kidney allograft via RT-PCR. We used both common assays (Emergency Use Authorizations approved) and research-only methods (optimized in-house) to detect for SARS-CoV-2 genetic material in a variety of biological tissues important in organ transplantation. Although larger studies are required to accurately identify the specific risk factors, in the current absence of detailed guidelines for organ procurement from SARS-CoV-2-positive donors, we believe our findings may help identify clinical scenarios conducive to safe organ transplantation, effectively expanding the supply of transplantable organs.

## DATA AVAILABILITY

The data that support the findings of this study are available on request from the corresponding author. The data are not publicly available due to privacy or ethical restrictions.

## DISCLOSURE

Michael Kueht has received grant funding from CareDx and Advisor Board honorarium from CareDx, Inc. and Veloxis Pharmaceuticals. Muhammad Mujtaba has received grant funding from CareDx.

## References

[1] Cucinotta D, Vanelli M. WHO declares COVID-19 a pandemic. Acta Biomed 2020;91:157–60. 32191675 10.23750/abm.v91i1.9397 PMC7569573

[2] U.S. Department of Health & Human Services . Summary of current evidence and information–donor SARS-CoV-2 testing & organ recovery from donors with a history of COVID-19. Health Resources & Services Administration 2022 (21 March 2022, date last accessed). https://optn.transplant.hrsa.gov/media/kkhnlwah/sars-cov-2-summary-of-evidence.pdf.

[3] Carugati M, Morlacchi LC, Peri AM, Alagna L, Rossetti V, Bandera A, et al. Challenges in the diagnosis and management of bacterial lung infections in solid organ recipients: a narrative review. Int J Mol Sci 2020;21:1221. 10.3390/ijms21041221 PMC7072844 32059371

[4] Heinz N, Griesemer A, Kinney J, Vittorio J, Lagana SM, Goldner D, et al. A case of an infant with SARS-CoV-2 hepatitis early after liver transplantation. Pediatr Transplant 2020;24:e13778. 32559354 10.1111/petr.13778 PMC7323125

[5] Hologic Inc. Fact Sheet For Patients, Panther Fusion SARS-CoV-2. www.fda.gov. 2022 (21 March 2022, date last accessed). https://www.fda.gov/media/136155/download

[6] Abbott . Alinity m SARS-COV-2 Assay. Chicago, IL, USA: Abbott, 2022. www.molecular.abbott (04 March 2022, date last accessed). https://www.molecular.abbott/us/en/products/infectious-disease/alinity-m-sars-cov-2-assay

[7] Yuan X, Theruvath AJ, Ge X, Floerchinger B, Jurisch A, García-Cardeña G, et al. Machine perfusion or cold storage in organ transplantation: indication, mechanisms, and future perspectives. Transpl Int 2010;23:561–70. 20074082 10.1111/j.1432-2277.2009.01047.x

[8] Taylor MJ, Baicu SC. Current state of hypothermic machine perfusion preservation of organs: the clinical perspective. Cryobiology 2010;60:S20–35. 19857479 10.1016/j.cryobiol.2009.10.006 PMC2891866

[9] GeneXpert . Xpert Xpress SARS-CoV-2 - Instructions for Use. Sunnyvale, CA, USA: Cepheid, www.fda.gov. April 2022. (21 March 2022, date last accessed). https://www.fda.gov/media/136314/download

[10] Kami W, Kinjo T, Arakaki W, Oki H, Motooka D, Nakamura S, et al. Rapid and simultaneous identification of three mutations by the Novaplex™ SARS-CoV-2 variants I assay kit. J Clin Virol 2021;141:104877. 34134034 10.1016/j.jcv.2021.104877 PMC8164504

[11] Ortho-Clinical Diagnostics, Inc. Serology Test Evaluation Report for “VITROS Immunodiagnostic Products Anti-SARS-CoV-2 IgG Reagent Pack”. www.fda.gov. Silver Spring, MD, USA: Food and Drug Administration (FDA), 3 October, 2020 (21 March 2021, date last accessed). https://www.accessdata.fda.gov/cdrh_docs/presentations/maf/maf3371-a001.pdf.

[12] Thwe PM, Maiyo E, Ren P. Abbott ID now COVID-19 assay performance: a year in review. Diagn Microbiol Infect Dis 2021;101:115536. 34634713 10.1016/j.diagmicrobio.2021.115536 PMC8418095

[13] Thwe PM, Ren P. How many are we missing with ID NOW COVID-19 assay using direct nasopharyngeal swabs? Findings from a mid-sized academic hospital clinical microbiology laboratory. Diagn Microbiol Infect Dis 2020;98:115123. 32673978 10.1016/j.diagmicrobio.2020.115123 PMC7334653

[14] Merola J, Schilsky ML, Mulligan DC. The impact of COVID-19 on organ donation, procurement and liver transplantation in the United States. Hepatol Commun 2020;5:5–11. 33043228 10.1002/hep4.1620 PMC7537114

[15] Knechtle SJ, Marson LP, O'Callaghan J, Leuvenink H, Friend P, Pleog R. Chapter 9. In: Kidney Transplantation: Principles and Practice. Philadelphia, PA: Elsevier, 2020, 130–41.

